# Transitioning between clinical and academic practice from the perspectives of clinical academic trainees, academic training programme directors and academic supervisors: a mixed methods study

**DOI:** 10.1186/s12909-025-06803-w

**Published:** 2025-02-13

**Authors:** Ruth Payne, Isabella Frejah, Elizabeth Abbey, Rosemary Badcoe, Brigitte Delaney, Caroline Mitchell

**Affiliations:** 1 Division of Clinical Medicine, School of Medicine and Population Health, University of Sheffield, Sheffield, England; 2 Sheffield Teaching Hospitals NHS Foundation Trust, Sheffield, England; 3 Clinical Academic Training Team, University of Sheffield, Sheffield, England; 4 Academic Unit of Primary Medical Care, University of Sheffield, Sheffield, England; 5 Primary Care, Faculty of Medicine and Health, University of Keele, Keele, England

**Keywords:** Out of Programme for Research (OOPR), Supported Return to Training (SuppoRTT), Integrated Academic Training (IAT)

## Abstract

**Background:**

The career pathway of clinical academics in the UK is challenging. To pursue academic endeavors, trainees often undertake approved time ‘Out of Programme for Research’ (OOPR), a standalone research fellow post or join an ‘Integrated Academic Training’ pathway. Time out of training may impact their clinical skills, confidence and competency. The aim of this qualitative study was to explore the challenges associated with returning to clinical training after prolonged leave for academic trainees.

**Methods:**

Stakeholders were clinical academic trainees and supervisors within the Yorkshire and Humber region of England, and training programme academic leads from universities across England. Qualitative data-analyses of verbatim recorded data from three focus groups and 12 individual telephone interviews were conducted within an a priori framework.

**Results:**

Returning to a high-stress environment with a perceived lack of specialty-level advocacy and support, feeling isolated from peers, struggling to balance competing demands, meet clinical and academic expectations and managing clinical deskilling in a trainee’s return to clinical training were common experiences described by stakeholders. There was a lack of recognition from academic leads, however, on the impact of such challenges on the trainees’ subsequent ability to successfully integrate their clinical and academic careers. Various solutions were identified by stakeholders to overcome such barriers, including a normalised, phased, individualised supported return to work and capacity building for supervisors.

**Conclusions:**

There is an apparent disconnect between the clinical and academic world, with clinical academic trainees stuck between the two, being pulled by each, feeling like they are not quite meeting the expectations of either. Time away from training for trainees on OOPR is often longer than for other reasons for time out of programme (typically 3–4 years if completing a doctoral degree). Given the importance of clinical academics in bridging clinical research and practice, and the concerns recently raised about the rate of attrition of clinician scientists within the NHS, it is of the utmost importance that clinical academic trainees are supported throughout all stages of their careers.

**Supplementary Information:**

The online version contains supplementary material available at 10.1186/s12909-025-06803-w.

## Background

There are approximately 70,000 trainee doctors undergoing post-graduate specialty training programmes across the United Kingdom. At any given time, it has been estimated that 10% of trainee doctors in England have approved time out of programme [1, 2] for reasons including parental leave, health and wellbeing, career breaks, to participate in courses outside of training programme, or increasingly, to undertake academic research [3]. In addition to those trainees taking approved ‘out of programme’ time, there are an increasing number taking time between training programmes, such as the ‘FY3’ year, which allows doctors to undertake a variety of roles, including as clinical research fellows for a year. The proportion of doctors taking an FY3 year rose from 10% in 2010 to 65% in 2019 [4].

Establishing and maintaining a clinical academic career is challenging: clinicians have to balance post-graduate training, with the required assessments and milestones, and the stress of clinical service alongside research and teaching endeavors [5]. Emerging evidence suggests that time away from clinical practice adversely impacts a health professional’s clinical skills, confidence and competency [6]. Length of time out of clinical practice has been identified as a key factor influencing the return to practice, with a longer time away associated with poorer clinical performance [7, 8]. This is of particular importance to the clinical academic trainee (CAT) workforce as ‘Out of Programme for Research’ (OOPR) schemes tend to be longer than other approved time out of training, often for three years or longer, for those completing a doctoral degree.

Given the unique position that clinical academics hold in bridging clinical research and practice, it is of the utmost importance that CATs are supported throughout all stages of their careers. Various policies have been developed and implemented to address the issues identified for trainees who take time out of training. The Academy of Medical Royal Colleges established Return to Practice Guidance [7], which has been adapted by some specialties (e.g., paediatrics, anaesthetics) [9, 10] to try to promote a smooth transition in and out of clinical practice. They recognise that trainees will have different needs upon their return to practice, and reflect an individualised, bespoke support approach. This aligns with the Supported Return to Training (SuppoRTT) initiative established by Health Education England in 2017 to ensure the safe and confident return to training after a period of absence, which includes a variety of training and learning opportunities, ideally adapted to the individual trainee.

Although recent evaluations of SuppoRTT programmes indicate that some aspects of the programmes are reported to be helpful (e.g. pre-return planning meetings), several areas in need of improvement have been identified [11]. One study evaluating SuppoRTT for General Practice trainees in Wessex, for example, identified the need for more wellbeing support and increased flexibility on return to clinical practice [12]. Additionally, most programmes have focussed on parental-leave or long-term illness reasons for being out of training, and there has been little bespoke support for academic trainees.

In response to the launch of SuppoRTT, we co-developed and evaluated a programme of work to support the successful transition for CATs between clinical and academic training posts. This research-led intervention complements work in the Athena Swan group within the University of Sheffield to proactively enable diversity, inclusion and non-discriminatory career progression in clinical academia [13]. The following qualitative study reports on the initial phases of the programme, which aimed to explore the challenges associated with the return to clinical training after prolonged leave for academic trainees. These findings have been used to co-produce interventions with CATs to improve clinical-academic transitions in the Yorkshire and Humber area of the UK.

## Methods

The reporting of the qualitative study adhere to the Consolidated Criteria for Reporting Qualitative Research (COREQ) checklist [14]. Ethical approval was obtained by the University of Sheffield ethics committee (No. 024474). All participants provided written or verbal informed consent.

### Study design

This qualitative study sits within a wider programme of work undertaken by the authors to ascertain the issues faced by stakeholders (trainees, supervisors and academic leads) with the aim of informing future interventions to support transitions between academic and clinical practice for CATs [15]. Three focus groups and twelve individual semi-structured qualitative interviews were conducted to explore and collect data on stakeholder perceptions and experiences of the challenges and facilitators to successful transitions between academic and clinical career phases. Focus groups were chosen to capitalise on facilitated conversations between participants and their near peers [16, 17]. The focus groups were divided into separate CATs and supervisor groups to address concerns about power differentials with explanation of the confidentiality and anonymisation of data analysis when individual consent for participation was sought. Semi-structured individual telephone interviews were undertaken with ACTS and supervisors unable to attend the focus groups and a national sample of university academic training programme directors (TPD) across England [18].

### Data collection

Trained facilitators (C.M., B.D., R.P.)^1^ used a topic guide (Supplementary material- ‘Focus group and interview topic guide’, Supplementary Table 1), developed by the research team, with reference to a scoping literature review (unpublished) and the Athena Swan Principles [13], to facilitate focus group discussion and telephone interviews with CATs, clinical academic supervisors, and TPDs. Participants were asked about barriers, challenges, and facilitators around returning to training after time out of programme and discussed possible solutions to the barriers identified. Focus groups ran for approximately 60 min and interviews for 20–40 min. Both were recorded and later transcribed verbatim. Field notes taken during the focus groups and interviews were discussed with C.M. during a debrief. No responder triangulation took place. Additionally, structured telephone interviews using a topic guide were conducted with 7 individuals who had expressed an interest in the focus group event but had been unable to attend. Contemporaneous field notes were taken, which were subsequently summarised and analysed.

Semi-structured telephone interviews with clinical academic TPDs at English Medical Schools were conducted with reference to a topic guide developed by the research team (Supplementary material, ‘England Clinical Academic Training leads telephone interviews topic guide’). Participants were asked about barriers and facilitators for CATs following OOPR and any interventions at regional and national level that have been implanted to support transitions between academic and clinical training. As permission to record the interviews was not sought, contemporaneous field notes taken during interviews were summarised and analysed. The telephone interviews were conducted by experienced qualitative researchers able to interview and to take contemporaneous notes and summarise the key themes effectively. This phase of qualitative enquiry was supplementary to the in-depth transcribed data from focus group methodology used in the workshops- to test and explore these themes in other areas and contexts. We did not have access to telephone recording equipment, and the interviews could not be conducted face-to-face because they were geographically dispersed and accommodated busy clinical academic schedules - often times were changed at short notice.

### Data analyses

Qualitative data were analysed on NVivo [19] using an á priori framework [20] to investigate barriers, facilitators and solutions to address challenges in the return to clinical training after prolonged (six months or more) leave for CATs. The five key stages of Ritchie & Spencer [20] framework were utilised: familiarisation, identifying a thematic framework, indexing, charting, and mapping/interpretation. Data were initially coded into categories and subcategories by two authors (B.D. and I.F.) to develop a coding framework, which was discussed and agreed upon by the chief investigator (C.M.). The framework was applied to the remaining transcripts and field notes, with regular discussions between team members to allow for triangulation and to ensure consistency in interpretation.

De-identified demographic characteristics and survey data were summarised using simple descriptive statistics (frequency counts and percentages) with Microsoft Excel.

## Results

### Participants and recruitment

Twenty-four CATs, clinical/academic supervisors, and academic TPDs were identified through a mailout to the Yorkshire and Humber region. Maximum variety sampling by gender and specialty was used on established contact databases from the Clinical Academic Training hub at the University of Sheffield. Seventeen participants attended face-to-face focus groups at an off-site conference space, and seven participants completed telephone interviews in March 2019. Focus groups contained 4, 6 and 7 participants and facilitators (groups were not even in number as supervisors were in a separate group to trainees and individuals were allocated prior to attendance but some who had registered for the event did not attend).

Five clinical academic training leads from universities across England were identified from their institution’s website and completed telephone interviews in August 2019. The universities included Imperial College London, University College London, University of Leeds, University of Bristol, University of Manchester, and University of Leicester. Thirty additional Medical Schools were approached, but either did not respond or declined to participate. Reasons for refusal included that discussions would be more appropriate at their regional postgraduate deanery level.

### Focus group and interview results

Stakeholder characteristics for the focus groups and interviews can be found in Table 1.

**Table 1:** Stakeholder characteristics for focus groups and interviews

Characteristic		Count (%)
Age (*n* = 19)	20–30	1 (5%)
	31–40	8 (42%)
	41–50	8 (42%)
	51–60	2 (11%)
Gender (*n* = 17)	Male	8 (47%)
	Female	9 (53%)
Grade	Foundation Trainee	0
	Core Trainee (CT1/2)	0
	Early Specialist Training (ST1-4)	3 (16%)
	Late Specialist Training (ST5+)	7 (37%)
	Consultant/GP	9 (47%)
	Staff grade/locum post/other	0
Current post	Consultant (Clinical)	5 (21%)
	Consultant (Academic)	4 (17%)
	Non-Clinical academic supervisor	0
	Clinical Training Post	3 (13%)
	Clinical Academic Training Post (AFP/ACF/CL)	3 (13%)
	OOPR	4 (17%)
	OOPE	0%
	Academic training leads	5 (21%)

*Notes.* ACF = Academic Clinical Fellow; CL = Academic Clinical Lecturer; AFP = Academic Foundation Programme; CT = Core trainee; GP = general practitioner; OOPE = Out of programme experience; OOPR = Out of programme research; ST = Specialist trainee

Nine main themes coinciding with our a priori framework were identified. Through an iterative process informed by the in-depth exploration of novel perspectives from focus groups and interviews, sub-themes emerged. The main themes, sub-themes and five solutions to identified barriers with illustrative quotes have been summarised below.

### Individual level factors

#### Theme 1. Competing demands

Stakeholders described the difficulty experienced in managing conflicting academic and clinical demands. Trainees reported issues with finding time to regain clinical competencies during their academic training and managing the pressures of academic work, such as writing papers, completing funding applications or applying for clinical posts/academic fellowships, and performing their clinical role.

“*There’s a clock ticking for the next academic milestone and that is driving me and compromising my return to clinical work”* (Subtheme 1.2. Finding balance, trainee).

*I think for most people is the sense that I don’t know if I’m going to be academic successful or clinician successful* (Subtheme 1.2. Finding balance, trainee).

There was agreement between stakeholders of the importance of timely PhD/MD submissions to avoid the added pressure of completing their thesis in their ‘personal’ time.

“*…that isn’t the way the PhD is supposed to work*,* and it doesn’t lend itself very well to clinical academics because once you go back into your clinical work*,* if you haven’t written up and submitted at the end of your three years*,* then you’re in deep doodaas* (Subtheme 1.1. Time constraints, supervisor).

Trainees with families reported further pressure of finding family time amongst their clinical and academic commitments. There was a sense of distress from trainees that due to the competing demands on their time, they may not be able to excel both clinically and academically.

*“So*,* balancing the triad of clinical work*,* academic work and family seems like a big issue doesn’t it…I’ve certainly been told you can choose two out of the three!”* (Subtheme 1.2 Finding balance, trainee).

#### Theme 2. Trainee confidence

Trainees reported a sense of deskilling during their academic training and the loss of self-confidence in their clinical competence. This included both clinical skills and patient-related communication. Despite the stress and anxiety this initially caused trainees, however, there was agreement that the loss of self-confidence was short-lived.

“*What I found more difficult was the decision-making and not necessarily making the wrong decision but actually having confidence in my decision-making was what I struggled with most”* (Subtheme 2.1. Deskilling, trainee).

*“I have to say that most of my experience is that people reskill very quickly”* (Subtheme 2.1. Deskilling, supervisor).

There was further agreement between stakeholders that undertaking clinical work, such as locums and clinics, to maintain clinical skills during academic training boosts trainee self-confidence and facilitates a smooth transition to clinical training.

However, views on who is responsible for facilitating the maintenance of a trainee’s clinical skills during academic training differed between the stakeholders. Although some trainees reported that clinical time was embedded within their PhD programme, the majority indicated that they undertook clinical work at their own accord for fear of deskilling.

“*I locummed a lot when I was out of programme because I was terrified of going back and not being able to cannulate”* (Subtheme 2.2. Upkeeping clinical skills, trainee).

Of note, the academic training leads discussed that they were only responsible for academia, with maintenance of/training in clinical skills being the responsibility of the Deanery (Health Education England).

*“Any loss of clinical skills is a deanery issue*,* not a university matter*…” (Subtheme 2.2. Upkeeping clinical skills, clinical academic training lead).

#### Theme 3. Trainee relationships with colleagues, supervisors, and the associated expectations

Trainees and supervisors commented on the challenges associated with the expectations trainees place on themselves and perceive from colleagues to be a ‘good trainee’. There were expectations around being at a desired level of clinical competency, usually comparable to what they had been before leaving clinical training, with the implication that a returnee should be able to perform immediately to this level. This was particularly the case for trainees who were in a more senior position prior to time out of programme.

“*I think you do start jobs and you’re just expected to get going and get on with things”* (Subtheme 3.1. Expectations, trainee).

There was a sense from trainees and supervisors that academic work is not valued within the clinical sphere, with a lack of understanding from clinical colleagues on the nuanced demands of the clinical academic training pathway. Trainees reported feeling judged for pursuing academic endeavours, with some noting hints of jealousy from clinical peers, particularly if there was any indication of special treatment for CATs.

“*Now I’m back in clinical*,* nobody cares about anything academic… Which is wrong because your specialty should have a scholarly ethos”* (Subtheme 3.2. Academic work under-valued, trainee).

*“‘Oh wow*,* I wish I could have that’. So then it brews that jealousy almost of ‘I wish I had time and money given to me to go and do this beautiful diploma’*,* kind of thing”* (Subtheme 3.2. Academic work under-valued, trainee).

#### Theme 4. Health and wellbeing

Trainees reported that the challenges of returning to clinical training while simultaneously maintaining their academic careers were compounded by feelings of psychological distress: stress, anxiety and fatigue. They reported feeling isolated, with a lack of emotional support provided by colleagues and supervisors.

“*It wasn’t that I couldn’t remember the name of a drug*,* it was that I felt isolated and overwhelmed and I couldn’t necessarily ask for support when I felt I should be”* (Theme 4. Health and wellbeing, trainee).

Importantly, trainees were aware of the adverse psychological impacts of their heavy workloads and reported engaging in wellbeing-promoting activities to avoid burnout. *“There are times when I just feel physically and mentally exhausted and it helps that I’ve had some time off recently*,* just a break where I did nothing and that’s what it has to be*,* that I have to build in breaks into my time where I can work*,* work*,* work and then I have to build in the four or five days off where I do nothing and just spend some time with the family”* (Theme 4. Health and wellbeing, trainee).

### System level factors

#### Theme 5. Returning to a (un)supportive environment

A key factor reported to impact a trainee’s experience of returning to clinical training was whether they were returning to a supportive or unsupportive environment. When supervisors or specialties were found to be unsupportive, trainees often cited a lack of understanding for their academic and clinical needs.

*“They didn’t think about my clinical training needs or*,* and it wasn’t particularly helpful from a return to work perspective”* (Subtheme 5.1. Importance of understanding academic and clinical needs, trainee).

On the other hand, trainees returning to environments where time away for research is commonplace reported supportive and understanding supervisors, which eased their return to clinical practice. Notably, there were some cross-specialty differences in the level of support offered.

*“Our trainees have an educational supervisor who’s not just an educational supervisor really and they look after them for the whole stage of their training”* (Subtheme 5.1. Importance of understanding academic and clinical needs, supervisor).

Constraints on supervisory capacity was found to impact the supportiveness of a work environment for returning trainees. This included having a supervisory workforce that has the skillset to understand the nuanced needs of trainees as well as the capacity within their work week to attend to those needs. Factors such as the continuity of educational supervisors and having the capacity to keep in contact with trainees while out of programme was also mentioned.

*“Really what you need is*,* if it’s not the same person*,* which is probably a rare beast who understands both and wants to understand*,* you certainly need some mechanisms so that the two liaise properly”* (Subtheme 5.2. Lack of supervisory support and capacity, supervisor).

Trainees and supervisors agreed that one barrier to transitioning to clinical training is being placed on inappropriate rosters by roster managers who do not understand the clinical needs of the trainees. This included being placed on-call or night shifts on their first week(s) of their clinical training. This created a sense that the workplace was not supporting the return of trainees.

*“The biggest stumbling block is often rota-meisters…just stick you on a rota*,* you’re no.9 and you’re therefore working this weekend*,* whether you’re returning to training or you’re in the middle of your training*,* so I think that’s a huge issue we’ve let go”* (Subtheme 5.3. Rota issues, supervisors).

#### Theme 6. Clinical training

Another system-level barrier identified by trainees and supervisors was the lack of available training for upskilling, and the lack of funding to support training (where available). If trainees wanted to maintain their skills, such as attending a training day or shadowing at a clinic, they had to pay out of pocket or volunteer their time.

*“…for our [teaching days] when I went out of programme I found that I had to pay for them”* (Theme 6. Clinical training, trainee).

#### Theme 7. Forward planning

Trainees were not always aware of forward planning around rotas, on-calls, and shift patterns to allow them some say about which hospital they returned to and be aware of what clinical support would be appropriate. Trainees who had a supported return to work discussed the importance in having pre-return planning meetings with supervisors and TPDs.

All stakeholders agreed on the importance of a flexible, phased return to clinical training tailored to the needs of each trainee. Issues of funding the phased return were notable barriers to implementation.

*“But if I could have chosen it*,* perhaps a daytime shift where you’re a bit more supported and perhaps a bit of a shadowing period back where you just have a little bit more support*,* especially on the operating side*,* would have been better rather than night shift on your own coping with everything”* (Subtheme 7.2. Phased return, trainee).

*“It’s a great idea*,* I don’t know who the hell would fund a phased return for an academic*,* because they’ve had three or three and a half years’ salary from a grant and that will stop and the university is not suddenly going to go ‘well we’ll pay for the academic side of your phased return’”* (Subtheme 7.2. Phased return, supervisors).

In contrast, however, most of the academic clinical leads reported that their trainees handled the transition back to clinical training well, with no institution implementing explicit support for clinical academic trainees returning to clinical practice.

Many trainees further commented that they had to request a supported return to work– including a pre-return planning meeting with supervisors to discuss their needs, a phased return or training requirements. In some instances, such supports were not in place within the specialty, but trainees noted the willingness of specialties to provide a tailored return to work upon request.

*“So*,* there was a kind of return*,* you know*,* a kind of plan in place*,* it’s just that the only thing that surprised me is that you do need to force your way to get it*,* that’s what surprised me and that was a big shock”* (Subtheme 7.3. Requesting support, trainee).

#### Theme 8. Trainee awareness of available supports and resources

A common barrier reported by trainees and supervisors in accessing support systems when returning to work was the lack of knowledge and signposting around what supports were available.

“*I don’t know if I was just living on another planet*,* but I had no idea about any of these resources available to returning trainees*,* was never signposted to that at all”* (Theme 8. Trainee awareness of available supports and resources, trainee).

#### Theme 9. Environmental challenges

Trainees reported feeling less well supported day-to-day where the return was to an unfamiliar environment, with new or updated protocols and administrative systems (e.g., colour of forms, forms moving online). In contrast, coming back to a familiar service setting in terms of knowing colleagues and knowing the systems, was felt to be useful.

*“…because you know*,* you know the people*,* you know what their structure’s like so it felt easy going back and I felt welcomed back”* (Subtheme 9.1. New work environments, trainee).

### Solutions

#### Solution 1. Establishing a clear transition pathway between out of programme and return to clinical work

The first solution identified by stakeholders was establishing a clear transition pathway between out of programme and return to clinical work. The importance of ensuring a mutual understanding of expectations between returning trainees and supervisors was emphasised. Vital to the transition pathway was normalising a phased return to clinical training. This included phasing into nightshifts, on-calls, or full-time workload and having allocated time to complete mandatory administrative tasks. Of note, academic training leads suggested that there should be time allocated for academic work within the phased return (e.g., time set aside for writing up papers based on their PhD).

There was some discussion around whether the phased return to work should be formalized and mandated within a cross-specialty guideline. This included mandating minimal requirements (e.g., meetings with supervisors for forward planning) and designating what a phased return should look like based on the length of absence from clinical training. Some supervisors reported that mandating a phased return would increase the burden on trainees, while others noted it would unify processes across specialties.

“*We started to talk around the idea of having a very set rule around if you’ve had say over a set period of time*,* like six months*,* a year*,* then you have to go through a formalised sort of phased return process that doesn’t allow you to*,* say*,* start on the night shift on your first day back or*,* you know*,* you’re phased back into that slowly”* (Solution 1, trainee).

*“The big question that’s come from the trainees that I work with is should all this stuff be mandatory when you’re coming back? There’s a big push back against that because there’s a lot of thought that there’s already too much paperwork in the system and I should be able to design my own individualised return to work plan and if I don’t think I need any help then I shouldn’t need to access it and all this sort of stuff”* (Solution 1, supervisor).

#### Solution 2. Individualised return to work

Trainees and supervisors discussed the importance in ensuring that support for return to work was personalised, recognising that the clinical, academic and training needs for each trainee will differ.

*“The personalisation of whatever reintroduction is needed and that that should be done early and that that should include the TPD educational supervisor*,* academic supervisor and clearly the trainee and whatever other representative so that it’s really tailored”* (Solution 2, trainee).

#### Solution 3. Funding a supported return

To ensure a clear, supported and individualised transition to clinical work (solutions 1 and 2), stakeholders reported the need for a ‘return to programme clinical training budget’. This included supernumerary funding for phased return and funding to participate in clinical skills training sessions.

“*…a solution I would propose is that somehow or other*,* whether it comes from research money or comes from the university or comes from the deanery*,* I would love to see four weeks of paid supernumerary time for these academics”* (Solution 3, supervisor).

Of note, one supervisor commented that they would willingly fund clinical reengagement activities, but *“I’m not going to give you extra study leave so that you can finish a job that you should have finished within a deadline”*.

#### Solution 4. Importance of supervisors

Participants identified a key pastoral support role of active and sympathetic supervisors and TPDs in addition to their role in supervising integrated clinical and academic career progression. It was suggested that good relationships help facilitate the return to work, maintain links throughout the time out of programme, provide support and advice, and give reassurance and aid confidence.

*“Nurture your relationships*,* both your clinical team and your academic team*,* because they’re really*,* really important*,* everybody’s talking to each other and there was a career trajectory timeline from the get-go and then I think there’s a much better chance that that will be realised” (Solution 4*,* supervisor)*.

There was some discussion that clinical trainees should be assigned a supervisor that has experience of supervising clinical academic trainees and understands the pressures and expectations associated with the transition back into clinical work. To build the capacity of supervisory team, it was suggested that training should be provided to supervisors and TPDs about the specific requirements CATs need on their return to work.

“*Actually*,* you know*,* one solution is to provide more training to educational supervisors so they understand more about returning trainees… kind of support for educational supervisors to understand how to coach the trainee back in”* (Solution 4, trainee).

#### Solution 5. Additional supports

Additional supports discussed by trainees and supervisors included peer networks and support champions. The establishment of peer networks for CATs within a region were suggested as an informal support system. Support champions were suggested to help facilitate meetings with trainees and supervisors to identify needs and supports required.

## Discussion

Clinical academic trainees face various challenges when returning to clinical training. They are returning to a fast-paced, high-stress environment in which they are expected to meet service delivery requirements, while simultaneously expected to meet academic milestones. The clash of the academic and clinical worlds can leave clinical academic trainees unsupported, misunderstood, overworked and at risk of burnout. These trainees are often highly motivated, ambitious and have high expectations set on them– both by others and themselves. This can lead to misalignment between what the trainee feels and what they express, or what others (including supervisors) observe.

This study demonstrates that CATs experience the same fears and anxieties on returning to training as those reported by trainees who have taken time out for other reasons (e.g. parental leave) [21] with loss of confidence in skills/knowledge being the most commonly reported issue. Although there is increasing drive from academic institutions for doctoral students to submit theses within the fee-paying period, there is inevitably additional academic work for trainees after the end of their OOPR period– whether preparation of work for publication, additional grant/CAT post applications or teaching commitments. As evidenced, this may contribute to trainees feeling overwhelmed and pulled in different directions trying to satisfy the expectations of their academic and clinical supervisors. Allowing a phased return option for some individuals may be an option that would smooth this transition and reduce the stress of contradictory demands reported.

This research contributes to an area for which there is currently little published data. The strengths of this work include the prospective designs of research tools, integration of multiple stakeholder perspectives (trainees, supervisors and TPDs) and a framework which provided an overarching structure with processes that allowed emergent sub-themes to be explored deeply, driven by participants more than the researchers. Flexible qualitative data collection using focus groups and supplementary interviews enabled us to gather data from a wider number of stakeholders, across a wider geographical area than a single in-person workshop to host focus groups. This also allowed us to explore potential solutions to the problems identified in earlier work with an engaged group of CATs who had all experienced at least one return to training in the past.

The limitations of our research processes include biased sampling to those who have managed the transitions successfully as we included participants currently on OOPR or in CAT posts; those whose experiences had led to burnout or disillusionment with research would likely have been missed if they have left academia. In addition, although we separated trainees and supervisors in focus groups, those who had experienced the highest levels of stress or burnout may not have felt comfortable discussing those issues in such a forum. Conversely, the self-selection of trainees to attend these groups may bias the data towards trainees who had previously experienced a more difficult transition, or who were more anxious about returning to training than those who did not attend. The lack of recording of telephone interviews could also be viewed as a limitation - but this is an acceptable form of qualitative data collection commonly used in policy research. It is possible to include non-recorded data within qualitative studies and is methodologically acceptable. It is a particularly useful method once initial transcribed data has been collected and analysed [22].

Although clinical academic training pathways have been in place for 17 years, these unfortunately often do not meet the ideal of the ‘Integrated Training Programme’ they are described as, with trainees’ clinical roles poorly aligned to their academic interests or needs, instead being used to fill rota gaps, where clinical service provision is prioritised over a bespoke academic training programme. This leads to trainees feeling demoralised, experiencing more negative emotions associated with transitions between academic and clinical training blocks, and may discourage trainees from pursuing additional CAT posts if their working environment does not prioritise research. Despite the development of clinical academic training pathways, the proportion of clinicians who choose an academic career has fallen over time, with wider equalities in terms of gender and ethnicity differences than are seen in either clinical or academic settings alone [23, 24].

The SuppoRTT programme is an important development in recognising the difficulties trainees face returning to a clinical environment after a period of time out. Although academics make up a substantial number of trainees who take time out, there are limited resources in place within these programmes that are focused specifically on the needs of academic trainees. Within the Yorkshire and Humber region only 51% of eligible academic trainees took up any of the services offered by the SuppoRTT team (personal communication). Following the research described here Yorkshire and Humber SuppoRTT team agreed to fund academic SuppoRTT champions for the region– at the time of writing these are the only academic SuppoRTT champions in the country, to our knowledge. If increased engagement with the programme is desired, it is essential that these trainees feel as though their needs are being catered for, and that research is seen as worthwhile, rather than only the clinical aspects of training.

## Conclusion

More needs to be done to integrate academia and clinical training as the current disconnect contributes to additional stress and challenges for trainees who are already juggling competing demands. Similar challenges and reasons for clinical academic attrition have been published in work funded by some of the biggest research funders in the UK, with similar conclusions to ours, requiring collaborative working between universities and healthcare organisations to support and nurture clinical academics in order to improve their experiences [24]. The specific challenges of academic trainees who take time out of training programmes need to be addressed proactively, recognising the differences (as well as similarities) for these trainees in comparison with those taking time out for other reasons. Clinical academics are a key part of the academic and healthcare workforces, bridging clinical research and practice and contributing to improved care for patients through novel diagnostic and therapeutic developments, so failing to address the underlying causes for the loss of clinicians from this career pathway will have a substantial negative impact. Senior clinical academics who have navigated a similar path previously can provide support both within academic institutions and healthcare environments, as well as engaging with training programme providers. The implementation of academic SuppoRTT champions within the Yorkshire and Humber region is one of the ways we have sought to do this, based on our data.

## Electronic supplementary material

Below is the link to the electronic supplementary material.

Supplementary Material 1

## Data Availability

The datasets used and/or analyzed during the current study are available from the corresponding author upon reasonable request.

## Footnotes

1 Caroline Mitchell: MBChB, MD, Professor of General Practice Research and General Practitioner. Experienced senior qualitative researcher. Minimal prior relationship with stakeholders. Brigitte Delaney: BA. Trained qualitative researcher in clinical and medical education research. No prior relationship with stakeholders. Ruth Payne: MBChB, MSc, PhD, Senior Clinical Lecturer and Consultant Microbiologist. Minimal prior relationship with stakeholders.
