# Supplementary material for: Transitioning between clinical and academic practice from the perspectives of clinical academic trainees, academic training programme directors and academic supervisors: a mixed methods study
Source: BMC Med Educ. 2025 Feb 13;25:236. doi: 10.1186/s12909-025-06803-w (PMC11827248; doi:10.1186/s12909-025-06803-w)
Supplement: Supplementary file 1 — Supplementary Material 1 [file 12909_2025_6803_MOESM1_ESM.docx]

# Supplementary material

## Supplementary methods

### Focus group and interview topic guides

*Supplementary Table 1*. Focus group and interview topic guide

| Barriers | - On an individual level what do you identify as barriers or challenges to returning to training after time out of programme? - What are the organisational barriers or challenges? - What do you think are the five key barriers when returning to training? |
| --- | --- |
| Facilitators | - What do you that the facilitators to returning to training after time out of programme? Again thinking from individual and organisational perspectives - Can you think of any examples of good practices for trainees returning after time out of programme? - What do you think are the five key facilitators when returning to training? |
| Solutions | - We would like to spend some time thinking about and identifying possible solutions to the barriers identified. - [Please take some time to] brainstorm - [We would like you to] rank the solutions from most important to least important (up to ten) - How might the solutions work in practice and what might stop them from working? |

### England Clinical Academic Training leads telephone interviews topic guide

| SuppoRTT project name - relevant to clinical academic trainee time out of programme |
| --- |
| Organisation if different from above e.g. HEE or offered by employing trusts |
| Clinical specialty- Specialty specific or across all CAT specialties |
| Please provide a brief description of the project/ activity |
| What is the likely or perceived impact?  What do you see as the main outcomes of this activity? |
| Who else could we talk to about this case study who might be able to give us more detail or a different perspective? |
| What have you learnt from delivering this SuppoRTT CPD activity?  What in particular helped participants feel transitions between clinical and academic work were improved ? |
| What barriers were faced in identifying appropriate participants (CATs)  and enabling them to attend this?.  How did you overcome these barriers? |
| To what extent were doctors able to reflect on the tailored intervention to support CATS? |
| Have you written it up at all?  Could we see a copy? |
| Date implemented – How long ago? |
| Numbers if known  - i.e. how many doctors have benefited from this? |
| Format of sessions- ½ day; full day/ workshops with a taught component? |
| Is this CAT SuppoRTT CPD still continuing?  Are there any plans to develop it further or roll out wider?  What might you do differently in the future? |
| If we wanted to look at this example in more detail who do you advise we speak to?  Contact details for lead person |
| Are you happy for this to be written up as a case study?  Assuming yes, we can send you a copy for you to review and annotate if you wish. |
| Do you wish to identified in the case study or would you prefer it to be anonymised? |
